# Polygenic Risk Score for Alzheimer’s Disease Is Associated With Ch4 Volume in Normal Subjects

**DOI:** 10.3389/fgene.2019.00519

**Published:** 2019-07-10

**Authors:** Tao Wang, Zhifa Han, Yu Yang, Rui Tian, Wenyang Zhou, Peng Ren, Pingping Wang, Jian Zong, Yang Hu, Qinghua Jiang

**Affiliations:** ^1^School of Life Sciences and Technology, Harbin Institute of Technology, Harbin, China; ^2^Information Department, Jiangsu Singch Pharmaceutical Co., Ltd., Lianyungang, China

**Keywords:** Alzheimer’s disease, single nucleotide polymorphisms, polygenic risk score, Ch4 region, *APOE*

## Abstract

Alzheimer’s disease (AD) is a common neurodegenerative disease. *APOE* is the strong genetic risk factor of AD. The existing genome-wide association studies have identified many single nucleotide polymorphisms (SNPs) with minor effects on AD risk and the polygenic risk score (PRS) is presented to combine the effect of these SNPs. On the other hand, the volumes of various brain regions in AD patients have significant changes compared to that in normal individuals. Ch4 brain region containing at least 90% cholinergic neurons is the most extensive and conspicuous in the basal forebrain. Here, we investigated the relationship between the combined effect of AD-associated SNPs and Ch4 volume using the PRS approach. Our results showed that Ch4 volume in AD patients is significantly different from that in normal control subjects (*p*-value < 2.2 × 10^−16^). AD PRS, is not associated with the Ch4 volume in AD patients, excluding the *APOE* region (*p*-value = 0.264) and including the *APOE* region (*p*-value = 0.213). However, AD best-fit PRS, excluding the *APOE* region, is associated with Ch4 volume in normal control subjects (*p*-value = 0.015). AD PRS based on 8070 SNPs could explain 3.35% variance of Ch4 volume. In addition, the *p*-value of AD PRS model in normal control subjects, including the *APOE* region, is 0.006. AD PRS based on 8079 SNPs could explain 4.23% variance of Ch4 volume. In conclusion, PRS based on AD-associated SNPs is significantly related to Ch4 volume in normal subjects but not in patients.

## Introduction

Alzheimer’s disease (AD) is a complex and severe neurodegenerative disorder. It is characterized by progressive deterioration in cognition and behavior, which seriously affects people’s daily life (Hu et al., 2017; Jiang et al., 2017; Liu et al., 2018). Genetic factors can lead to 60–80% of AD risk (Lambert et al., 2010). The *APOE* gene is the strongest genetic risk factor for late-onset AD (Corder et al., 1993). Several existing AD genome-wide association studies (GWASs) have identified many common single nucleotide polymorphisms (SNPs) with relatively small effect size (Hindorff et al., 2009; Lambert et al., 2013). The combined effect of these SNPs could make a significant contribution to AD risk. The polygenic risk score (PRS) was described to depict quantitatively the combined effect of SNPs on disease risk (International Schizophrenia Consortium, 2009). It has been reported that PRS based on disease-related SNPs was associated with disease risk and can work as a predictor of disease risk (Escott-Price et al., 2015; Lupton et al., 2016; Escott-Price et al., 2017). In addition, several authors investigated the effect of PRS on both disease status and disease-associated phenotypes (also called endo-phenotype) (Harris et al., 2014; Marden et al., 2016; Axelrud et al., 2018). Axelrud et al. (2018) found AD PRS was an implication for memory performance and hippocampus volumes in early life. Harris et al. (2014) found there was no significant association between polygenic risk for AD and cognitive ability in non-demented older people. PRS for AD was utilized to predict memory decline in black and white Americans (Marden et al., 2016). Some studies have reported that the brain structure changes significantly in some nervous system disease compared to normal subjects by using magnetic resonance imaging (MRI) (Zhang et al., 2011; Alattas and Barkana, 2015; Mattavelli et al., 2015). In addition, brain-associated endo-phenotypes were commonly used to analyze the effect of disease-associated SNPs. Late-onset AD PRS was used to predict hippocampus function (Xiao et al., 2017). AD polygenic risk was proved to modulate precuneal volume (Li et al., 2018). Terwisscha van Scheltinga et al. (2013) found schizophrenia-associated genetic risk variants jointly modulate total brain and white matter volume by PRS approach.

Recently, a study demonstrated that basal forebrain degeneration precedes the cortical spread of AD pathology (Schmitz et al., 2016). There is the early pathological change of the nucleus basalis of meynert (NbM) in the basal forebrain (Grothe et al., 2012, 2013). Basal forebrain consists of magnocellular cholinergic cells and designated into Ch1–Ch4 according to the distribution difference of cholinergic neurons, with Ch4 corresponding to NbM (Mesulam et al., 1983). Ch4 region is the most extensive and conspicuous of Ch1–Ch4, containing more than 90% of cholinergic neurons (Mesulam et al., 1983). In fact, the Ch4 region provides the entire cortical surface with the single major source of cholinergic innervation (Mesulam et al., 1983). Ch4 region has plenty of functions, such as memory, attention, and modulation of the behavioral state (Gratwicke et al., 2013). Increasing studies have revealed that Ch4 region plays a major role in the function of memory (Butt and Hodge, 1995; Leanza et al., 1996; McGaugh, 2002). In addition, the Ch4 region and its cholinergic projections play an essential role in regulating a wide variety of attention functions (Voytko, 1996; McGaughy et al., 2002). Grothe et al. (2012) found atrophy of the cholinergic basal forebrain especially NBM (Ch4 region) in progressive AD. Previous studies have demonstrated that maximum 96% of Ch4 neuronal loss occurs in AD compared to normal control subjects (Whitehouse et al., 1981; Candy et al., 1983; Etienne et al., 1986). Volumetric MR imaging reveals that NBM (Ch4 region) significantly degenerates in AD patients compared with age-matched normal subjects (Hanyu et al., 2002). Teipel et al. (2011) discovered that the NBM (Ch4 region) cholinergic projection axons shrink in AD patients by high-resolution diffusion tensor imaging. Considering the early degeneration of Ch4 neurons in AD patients, we selected Ch4 brain region as an ideal candidate endo-phenotype to investigate the effect of AD-associated genetic risk variants.

It is well known that the *APOE* gene is significantly associated with AD risk. Therefore, in order to explore the *APOE* influence on AD PRS, PRS in this article is constructed based on AD-associated SNPs, excluding the *APOE* region and including the *APOE* region, respectively. This paper is aimed at exploring the relationship between AD PRS and Ch4 volume to answer following questions. Firstly, is there a significant difference of Ch4 volume between AD patients and normal control subjects? Secondly, is AD PRS significantly related with Ch4 volume in AD patients and normal control subjects, respectively?

## Materials and Methods

### Discovery Samples

Alzheimer’s disease GWAS summary data was obtained from the International Genomics of Alzheimer’s Project (IGAP) (Lambert et al., 2013). IGAP is a large two-stage study based on GWAS on individuals of European ancestry. In stage 1, IGAP performed a meta-analysis on four previous-published GWAS datasets containing 17,008 AD patients and 37,154 normal controls using 7,055,881 SNPs. In stage 2, 11,632 SNPs were genotyped and tested for association in an independent population consisting of 8,572 AD patients and 11,312 normal controls (Lambert et al., 2013). The stage 1 dataset is used to identify risk variants, their *P* values and corresponding odds ratios.

### Target Samples

Magnetic resonance imaging and genetic data used in this paper were available from the Alzheimer’s Disease Neuroimaging Initiative (ADNI) database ^1^. The ADNI was launched in 2003 as a public-private partnership, led by Principal Investigator Michael W. Weiner, MD. The primary goal of ADNI has been to test whether serial MRI, positron emission tomography (PET), other biological markers, and clinical and neuropsychological assessment can be combined to measure the progression of mild cognitive impairment (MCI) and early AD. We can obtain the SNP data and neuroimaging data of every participant at the same time in the ADNI database. In other words, both SNP and neuroimaging data are sampled from each participant in the ADNI database. We selected 108 AD patients and 182 normal control (NC) subjects according to sample diagnostic results. We removed four samples (099_S_4086, 027_S_1387, 116_S_1232, 037_S_4432) owing to their outliers of Ch4 volume. The remaining 106 AD patients (Supplementary Table S1) and 180 normal control subjects (Supplementary Table S2) were used as target samples for further analysis. All information on recruitment and diagnostic criteria could be reached on the ADNI website.

### MRI Analysis

Magnetic resonance imaging data were acquired according to a standardized protocol, which included a high-quality T1-weight, magnetization prepared rapid gradient echo (MP-RAGE) sequence (Jack et al., 2008). MP-RAGE acquisition parameters for one platform (Philips Medical Systems) are as follows: TR = 6.76 ms, TE = 3.11 ms, FA = 9°, matrix size = 256 × 256, slice thickness = 1.2 mm, number of slices = 170, voxel size x = 1.05 mm and voxel size y = 1.05 mm. Quality control of MRI data was performed at the Mayo Clinic based on centralized and standardized criteria (Jack et al., 2008).

All MRI data were transformed into NII files in the first place using MRIConvert software tool. All anatomical images were preprocessed by using the diffeomorphic anatomical registration through exponentiated lie algebra (DARTEL) in SPM12 (Ashburner, 2007). Basically, neuroimages were first segmented into the grey matter (GM), white matter (WM), cerebrospinal fluid (CSF), skull and soft tissue. Then, DARTEL was used to increase the accuracy of inter-subject alignment for generating a population template in montreal neurological institute (MNI) space. Finally, all GM neuroimages were normalized to MNI space based on the population template and smoothed with a Gaussian kernel of 8 mm, and they were subjected to modulation that depicted the tissue volumes. Voxel size for GM neuroimage was specified with 1.5 mm^3^. GM, WM and CSF volumes were available from the files containing segmentation parameters. The sum of these three tissues was computed as the total intracranial volume (ICV), and the sum of GM and WM volume was computed as the total parenchymal brain volume (TBV).

ROI for Ch4 in MNI space was achieved by using the SPM Anatomy toolbox (Eickhoff et al., 2005). Zaborszky et al. (2008) presented stereotaxic probabilistic maps of the magnocellular cell groups in human basal forebrain based on 10 postmortem brains, including Ch4 region. The ROI for Ch4 was created based on Ch4 probabilistic map. Because voxel size for the Ch4 ROI is 1 mm^3^, which is not consistent with smoothed and modulated GM neuroimage. It is necessary to co-register the Ch4 ROI with smoothed and modulated GM neuroimage. Co-registering Ch4 ROI and extracting ROI signals were performed utilizing DPABI software (Yan et al., 2016).

### Genetic Analysis

The genetic data were available from the ADNI webpage. ADNI participants were genotyped using the Illumina Omni 2.5M SNP arrays. The genetic data consist of 2,379,855 SNPs. We extracted 2,134,825 SNPs with rs or kgp prefix, which are located in 1–22 chromosomes. We performed a series of quality control procedures on these genetic data using PLINK tool set (Purcell et al., 2007). Firstly, individuals with more than 5% missing SNPs were removed. All participants approved the filter. Then, we removed 789,861 variants owing to minor allele frequencies of less than 0.02. Thirdly, 84,891 SNPs were taken away due to more than 1% missing genotypes. Next, we removed 2,597 variants according to Hardy-Weinberg exact test at a specified significant threshold of 1 × 10^−6^. Finally, in order to remove SNPs in linkage disequilibrium, 1,024,426 SNPs were pruned according to a pairwise *R*^2^ cutoff of 0.25 and a window of 50 SNPs with shifting five SNPs at every step (Terwisscha van Scheltinga et al., 2013). In the end, 233,050 variants with rs or kgp prefix were selected. 76,312 of 233,050 variants were available in the AD summary dataset. The genomic location for *APOE* gene is chr19: 45,409,011 – 45,412,650 (GRCh37/hg19). There are 11 SNPs with a 70 kb region which surround the *APOE* gene (rs1871047, rs11879589, rs387976, rs6859, rs283814, rs157582, rs405509, rs439401, rs445925, rs3760627, rs204479). We obtained 76,301 SNPs, excluding the *APOE* gene, and 76,312 SNPs, including the *APOE* gene, for subsequent analysis.

### Statistical Analysis

Individual age was computed as study date minus birth date. ICV was adjusted for age and gender. TBV, GM volume, WM volume and Ch4 volume were corrected for age, gender and ICV using linear regression in total groups. The correction method was described by Terwisscha van Scheltinga et al. (2013). Briefly, non-standard residual of volume for every participant could be obtained by linear regression. Then, the sum of non-standard residue of volume, intercept and$\sum_{\text{i}=1}^{\text{m}}{\mathrm{beta}}_{\text{i}}\times {\mathrm{mean}}_{\text{i}}$ was calculated as corrected volume, where m refers to the number of the covariate, *beta*_i_ represents the regression coefficient of covariate *i*, and *mean*_i_ denotes the mean of covariate *i*. All adjusted brain volumes are normally distributed in the total groups, AD patients and normal control subjects, respectively.

Polygenic risk score model is described by International Schizophrenia Consortium (2009). Every SNP has a corresponding *P* value for its association with AD. Basically, for each SNP, the variant risk score is calculated by multiplying the risk allele number (0, 1, 2) with the corresponding effect size, by the logarithm of the odds ratio. For each participant, the PRS is summed on all SNPs with *P* value below a threshold, *P*_T_. PRS is calculated at a series of *P* value thresholds, e.g., *P*_T_ = 0.0001, 0.0002, …, 0.05, …, 0.1, …,0.5. The *P* value threshold, *P*_T_, with the largest *R*^2^ is the most predictive cutoff. We calculated the PRS using a lower bound of *P* = 0, an upper bound of *P* = 0.6 and an increment of 0.0001 by PRSice software (version 1.25) (Euesden et al., 2015). PRSice can calculate PRS at a great number of cutoffs, apply PRS and plot the results of PRS.

The first ten principal components of population structure for AD patients and normal control subjects were achieved in PLINK software using the multidimensional scaling plot option (Purcell et al., 2007). And the number of non-missing SNPs used for scoring and inbreeding coefficient for AD patients and normal control subjects were also calculated in PLINK using the het option (Purcell et al., 2007). *APOE* status is coded as 0, 1, or 2, according to the number of *APOE* ε4. We performed linear regressions using Ch4 volume as an outcome variable in AD patients and normal control subjects, respectively, and the number of non-missing SNPs, inbreeding coefficient, the first ten population structure components and *APOE* status were as covariates. *R*^2^ was compared with a model only containing these covariates and a model containing these covariates and PRS. The difference in *R*^2^ between the two models is used to measure variance explained by PRS. These regression analyses were performed using PRSice (Euesden et al., 2015).

Gender difference between AD patients and normal control subjects is examined by the chi-square test in SPSS (version 22; IBM). Welch *t*-test is applied to examine brain volume and age difference between two groups using the R script. The *p*-value < 0.05 is considered statistically significant in this paper.

**Table 1:** Demographic information.

	AD patients	NC subjects	Significance
Participants	106	180	ns
Gender (M/F)	59/47	85/95	*p*-value = 0.1681
Age in Years (SD)	77.81 (7.2507)	76.90 (6.6234)	*p*-value = 0.2952
Participants with APOE ε4	77	43	ns
Intracranial volume in L (SD)^a^	1.4329 (0.1027)	1.4351 (0.1102)	*p*-value = 0.8633
Total brain volume in L (SD)^b^	0.9051 (0.0674)	0.9963 (0.0615)	*p*-value < 2.2 × 10^−16^
Gray matter volume in L (SD)^b^	0.5112 (0.0651)	0.5846 (0.0455)	*p*-value < 2.2 × 10^−16^
White matter volume in L (SD)^b^	0.3940 (0.0411)	0.4117 (0.0354)	*p*-value = 0.0002815
Ch4 volume (SD)^b^	0.2430 (0.0341)	0.3097 (0.0278)	*p*-value < 2.2 × 10^−16^

*F, female; M, male. ^a^Adjusted for age and gender. ^b^Adjusted for age, gender and intracranial volume.*

## Results

### Statistical Analysis of Brain Volume

**FIGURE 1:**
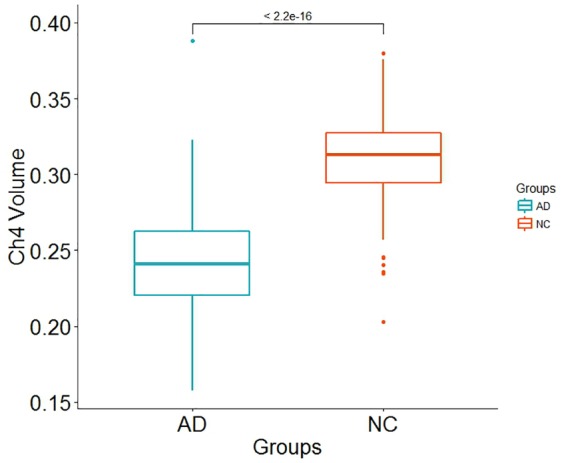
Box plot for Ch4 volume difference between AD patients and normal control subjects.

Demographic information is shown in Table 1. There is no significant differences in age (*p*-value = 0.2952) and in gender distribution (*p*-value = 0.1681) between AD group and normal control group. The number of participants with *APOE* ε4 in AD patients and normal control subjects is 77 and 43, respectively. In addition, it does not seem to make a difference in intracranial volume corrected for age and gender between the two groups (*p*-value = 0.8633). Total brain volume corrected for age, gender and intracranial volume in normal control subjects is larger than that in AD patients (*p*-value < 2.2 × 10^−16^). Our results indicated that both GM and WM volume adjusted for age, gender and intracranial volume in AD patients are smaller than that in normal control subjects (*p*-value < 2.2 × 10^−16^ and *p*-value = 0.0002815, respectively). In addition, Ch4 volume corrected age, gender and intracranial volume in AD patients is smaller than that in normal control subjects. Most importantly, there is a significant difference in Ch4 volume between AD patients and normal subjects (*p*-value < 2.2 × 10^−16^; Figure 1).

### The AD Polygenic Risk Score Is Not Associated With Ch4 Volume in AD Patients

**FIGURE 2:**
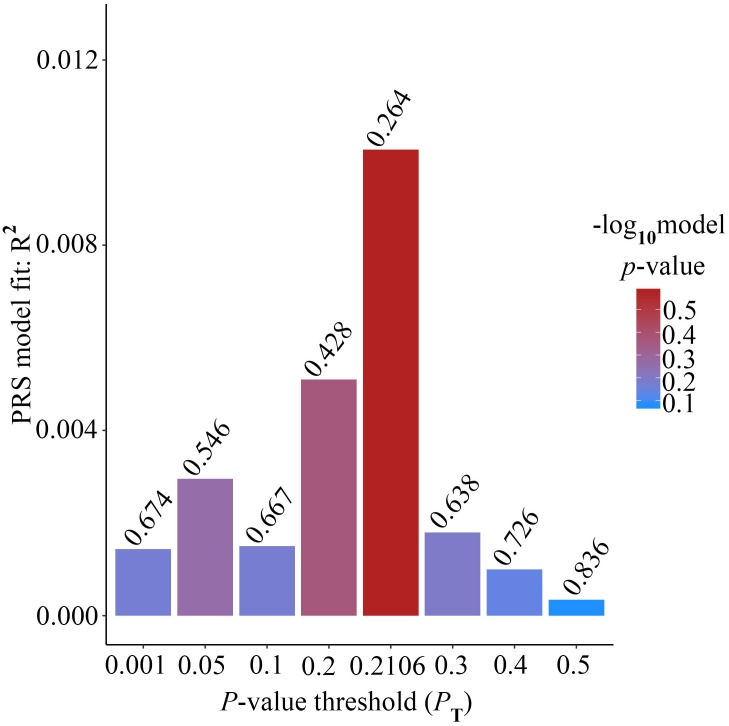
Bar plot showing at broad *P* value thresholds for AD PRS, excluding the *APOE* region, predicting Ch4 volume in AD patients, including a bar for the best-fit PRS from the high-resolution run.

**FIGURE 3:**
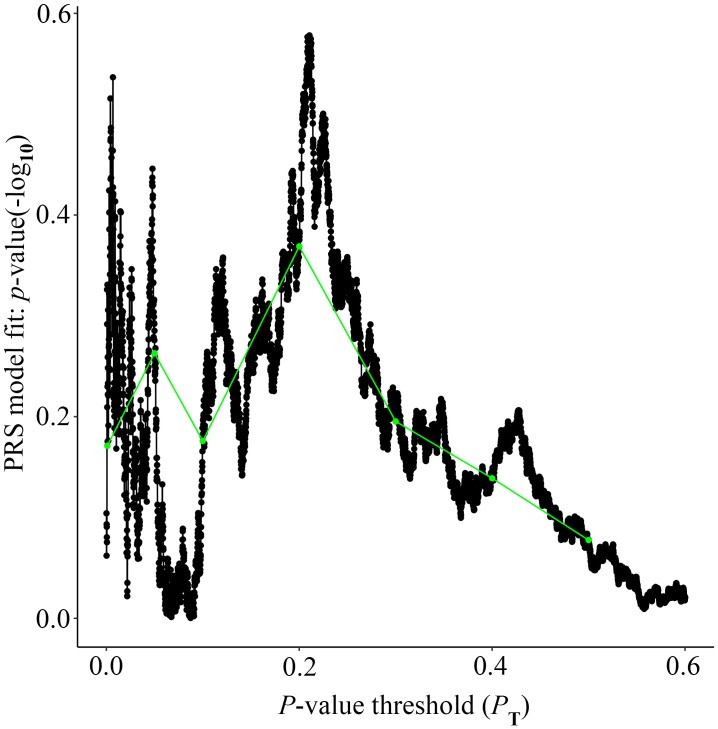
High-resolution plot for AD PRS, excluding the *APOE* region, predicting Ch4 volume in AD patients. The thick line connects points at the broad *P* value thresholds of Figure 2. The best-fit PRS is at *P*_T_ of 0.2106.

Alzheimer’s disease PRS based on AD-associated SNPs, excluding the *APOE* region, was used to predict Ch4 volume in AD patients using linear regression. There is no significant relationship between AD PRS and Ch4 volume at the different *P* value cutoffs (*P*_T_ = 0.001, 0.05, 0.1, 0.2, 0.3, 0.4, 0.5), because of all *p*-value of PRS model (*p*-value = 0.674, 0.546, 0.667, 0.428, 0.638, 0.726, 0.836) > 0.05, according to the PRS bar plot (Figure 2). On the basis of high-resolution PRS plot (Figure 3), the best-fit *P* value threshold for the PRS model is 0.2106. However, the *p*-value of PRS model at the best-fit cutoff is 0.264. These high-resolution scores indicate that the results from the broad *P* value cutoff of Figure 2 are not false negatives due to the small number of cutoff considered. The PRS base on AD-associated SNPs, excluding the *APOE* gene, is not related with Ch4 volume in AD patients. In addition, AD PRS, including the *APOE* gene, was utilized to predict Ch4 volume in AD patients. According to bar plot of PRS results (Supplementary Figure S1) and high-resolution plot (Supplementary Figure S2), the best-fit *P* value threshold for PRS model is 0.0068, and the *p*-value of PRS model at *P*_T_ = 0.0068 is 0.213. AD PRS, including the *APOE* gene, is also not related to Ch4 volume in AD patients. Therefore, AD PRS is not associated with Ch4 volume in AD patients. And AD PRS could not successfully measure Ch4 volume in AD patients.

### The AD Polygenic Risk Score Is Significantly Associated With Ch4 Volume in Normal Control Subjects

**FIGURE 4:**
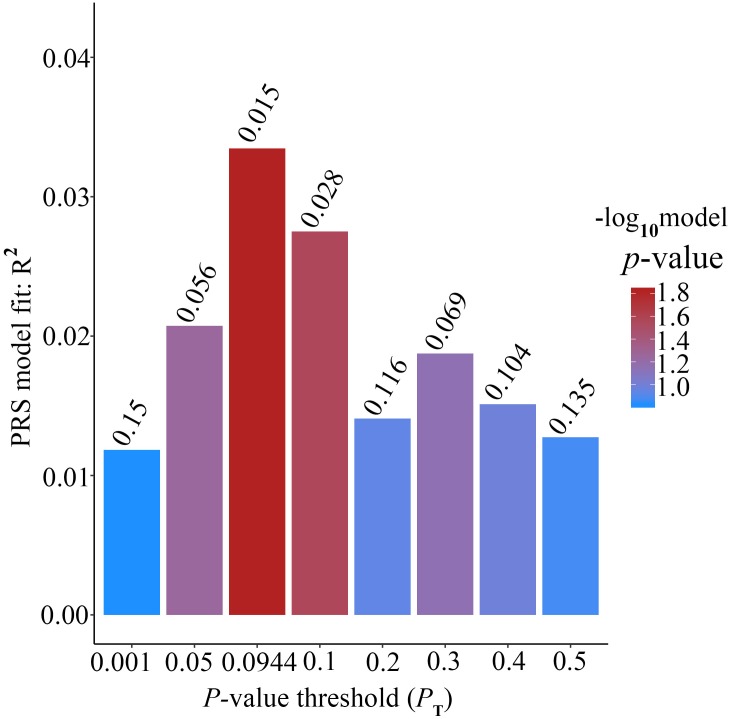
Bar plot showing at broad *P* value thresholds for AD PRS, excluding the *APOE* region, predicting Ch4 volume in normal control subjects, including a bar for the best-fit PRS from the high-resolution run.

**FIGURE 5:**
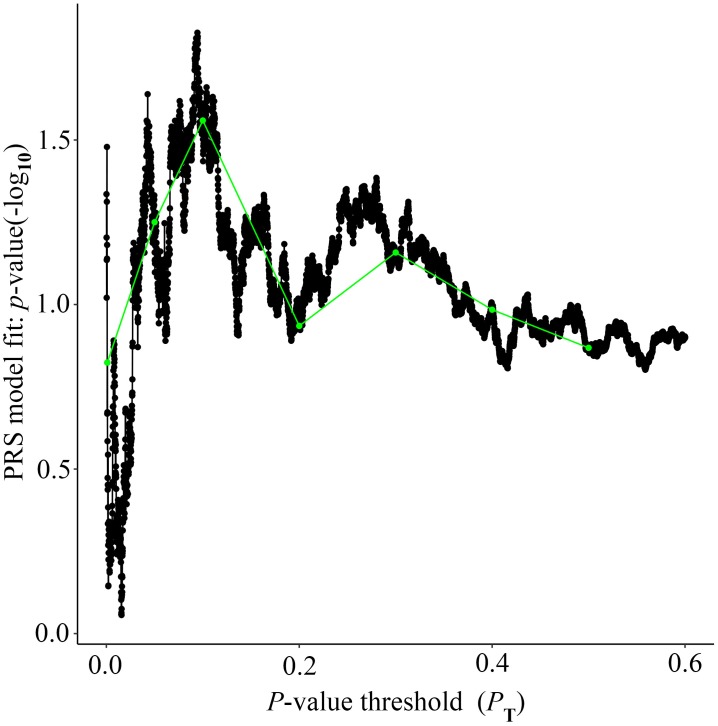
High-resolution plot for AD PRS, excluding the *APOE* region, predicting Ch4 volume in normal control subjects. The thick line connects points at the broad *P* value thresholds of Figure 4. The best-fit PRS is at *P*_T_ of 0.0944.

Alzheimer’s disease PRS based on AD-associated SNPs, excluding the *APOE* region, was used to predict Ch4 volume in normal control subjects. According to bar plot of PRS results (Figure 4), the *p*-value of the PRS model at *P* value threshold of 0.1 is 0.028. There is a significant relationship between AD PRS and Ch4 volume in normal control subjects at *P* value threshold of 0.1. On the basis of the high-resolution plot for PRS results (Figure 5), the best threshold for PRS model is 0.0944, the *p*-value of the PRS model is 0.015. There are 8070 SNPs (Supplementary Table S3) with their *P* value < 0.0944. AD PRS based on 8070 SNPs could explain 3.35% variance of Ch4 volume in normal control subjects. When *P* value threshold is more or less than the best *P* value threshold (*P*_T_ = 0.0944), the *p*-value of the PRS model will become greater than 0.015. When AD PRS contains more or fewer SNPs, the ability to account for the variance of Ch4 volume will decrease. AD PRS based on 8070 SNPs could act as a reliable measure for Ch4 volume in normal control subjects. In other words, AD PRS based on 8070 SNPs, excluding the *APOE* gene, is related to Ch4 volume in normal control subjects. Moreover, AD PRS, including the *APOE* gene, was used to predict Ch4 volume in normal control subjects. According to bar plot of PRS results (Supplementary Figure S3) and high-resolution plot (Supplementary Figure S4), the best-fit *P* value threshold for PRS model is 0.0944, and the *p*-value of PRS model at *P*_T_ = 0.0944 is 0.006. There are 8079 SNPs with their *P* value < 0.0944. AD PRS based on 8079 SNPs could explain 4.23% variance of Ch4 volume in normal control subjects. In other words, AD PRS based on 8079 SNPs, including the *APOE* gene, is significantly related to Ch4 volume in normal controls. Therefore, AD polygenic risk score is significantly associated with Ch4 volume in normal control subjects.

## Discussion

Alzheimer’s disease is a complex and polygenic disease. Current studies have demonstrated that many genetic variations are associated with AD. These genetic variations may be beneficial to understand the mechanism of AD to some extent. On the other hand, some brain regions associated with AD atrophy in AD patients by structural MRI technology. However, the details of association between some brain regions and genetic variation is still unknown. If we know this kind of detailed association, we could further get the regulatory relationship between genetic variation and brain region, which will provide valuable insights into disease mechanism, prevention and treatment. Ch4 brain region is associated with memory and cognition functions. Therefore, it is very important and necessary to analyze the association between genetic variation and Ch4 brain region.

The Ch4 brain region contains the largest, most hyper-chromic and polymorphic neurons in the basal forebrain, which supplies the single major source cholinergic innervation to the entire cortical surface (Mesulam et al., 1983). Ch4 volume could act as a phenotype associated with Alzheimer’s disease. In this article, we investigated the relationship between the combined effect of SNPs and Ch4 volume by using PRS. Our results indicated that the Ch4 volume in AD patients is smaller than that in normal control subjects, and there is the significant difference between the two groups (*p*-value < 2.2 × 10^−16^), which is consistent with the previous conclusions (Grothe et al., 2012, 2013; Schmitz et al., 2016). In addition, AD PRS, excluding or including *APOE* gene, is not linked with Ch4 volume in AD patients. However, AD PRS, excluding or including *APOE* gene, is significantly associated with Ch4 volume in normal control subjects. AD PRS could work as a reliable measure for Ch4 volume in normal control subjects.

Many studies found up to 96% of Ch4 neuronal loss in AD patients (Whitehouse et al., 1981; Candy et al., 1983; Etienne et al., 1986). AD PRS, excluding or including *APOE* gene, cannot measure successfully Ch4 volume in AD patients. This may be because Ch4 brain region in AD patients have shrunk severely so that there is no difference of Ch4 volume. Therefore, AD PRS, excluding or including *APOE* gene, may not be a suitable way to measure Ch4 volume in AD patients.

Many studies investigated AD-associated variants in biomarker measurements among healthy subjects using polygenic score approach (Small et al., 2000; Reiman et al., 2004; Filippini et al., 2009; Sheline et al., 2010; Sabuncu et al., 2012; Mormino et al., 2016). Sabuncu et al. (2012) found that the polygenic risk score was correlated with AD-specific cortical thickness in clinically normal human individuals, even after controlling for *APOE* genotype and other factors. AD genetic risk score can be used to predict the thinning of hippocampus complex sub-regions in normal older subjects (Harrison et al., 2016). Elizabeth et al. discovered that higher AD PRS was associated with smaller hippocampus volume in the younger healthy group (Mormino et al., 2016). The influences of common genetic risk variants are detectable among healthy subjects and may begin in early life (Mormino et al., 2016). Furthermore, some evidence reveals that AD-specific atrophy patterns can be identified before cognitive impairment (Csernansky et al., 2005; Jagust et al., 2006). In this study, AD PRS is significantly associated with Ch4 volume in normal control individuals. Our primary analysis suggests this association could be explained by a genetic modulation of neuro-degeneration, which is consistent with the interpretation of Sabuncu et al. (2012). This result agrees that AD-associated atrophy rates accelerate before the beginning of cognitive impairment (Mori et al., 2002; Schott et al., 2010; Andrews et al., 2016). AD PRS, excluding the *APOE* gene, at best-fit *P* value threshold (*P*_T_ = 0.0944) is significantly associated with Ch4 volume in normal controls. The *p*-value of PRS model at *P*_T_ = 0.0944 is 0.015. AD PRS based on 8070 SNPs could explain 3.35% variance of Ch4 volume. We further obtained 5397 genes of index 8070 SNPs from the dbSNP database. There are 3163 SNPs which do not have corresponding gene. 4452 SNPs have a unique corresponding gene. The rest of 455 SNPs have more than one gene. Then, we downloaded gene expression (transcripts per million, TPM) of brain nucleus accumbens (basal ganglion) tissue from Genotype-Tissue Expression (GTEx) database. We found that TPM of 3807 genes among 5397 genes is more than 0, which is about 70.54%. TPM of 3205 genes is greater than 0.5 (59.38%) and TPM of 2959 genes is more than 1 (54.83%). We will further validate these genes using biological experiments in the following studies. Furthermore, AD PRS, including *APOE* gene, at best-fit *P* value threshold is dramatically related with Ch4 volume in normal controls (*p*-value = 0.006). In addition, AD PRS based on 8079 SNPs could explain 4.23% variance of Ch4 volume. AD PRS including other nine SNPs in *APOE* gene could explain more variance of Ch4 volume (rs1871047, rs387976, rs6859, rs283814, rs157582, rs405509, rs439401, rs3760627, rs204479).

In this study, we investigated the relationship between AD-associated SNPs and Ch4 volume using PRS method. The polygenic risk score combines the weak effect of every candidate SNP in an additive model (International Schizophrenia Consortium, 2009). A great number of studies explore disease-associated genetic variants in disease status and disease-associated phenotypes (Small et al., 2000; Reiman et al., 2004; Filippini et al., 2009; Sheline et al., 2010; Sabuncu et al., 2012; Harris et al., 2014; Marden et al., 2016; Mormino et al., 2016; Axelrud et al., 2018). PRS model can capture nearly all common genetic risk for AD (Escott-Price et al., 2017). In fact, PRS cannot capture rare genetic risk variants and gene-gene interactions (Sabuncu et al., 2012; Escott-Price et al., 2017). In addition, there are some genetic risk variants contributing to Ch4 volume but without effect on AD, and AD PRS cannot capture. Lastly, some environmental factors may result in the change in brain volume, such as drugs (Navari and Dazzan, 2009; Moncrieff and Leo, 2010; Ebdrup et al., 2013). In future research, more sophisticated models considering these above factors should be constructed.

Considering that PRS based on AD-associated SNPs, excluding or including the *APOE* region, is associated with Ch4 volume in normal control subjects but not in AD patients. That is possibly because disease status severely changes the Ch4 volume to some extent (Whitehouse et al., 1981; Candy et al., 1983; Etienne et al., 1986). In conclusion, PRS based on AD-associated genetic risk variants is significantly associated with Ch4 volume in normal control subjects but not in AD patients.

Alzheimer’s Disease Neuroimaging Initiative database is a very canonical dataset for AD. Many scholars all over the world make their contributions to the mechanism of AD based on mining the ADNI dataset. We find the association between AD PRS and Ch4 brain volume based on the 180 normal control subjects download from the ADNI database. We want to replicate this result in another independent dataset. Therefore, we divided 180 normal subjects into several subsets.

There are 136 ADNI 2 stage normal subjects, 29 ADNI GO stage normal subjects and 15 ADNI 1 stage normal subjects among 180 normal subjects according to the diagnose information. We utilized 136 normal subjects as a discovery dataset and 29 normal subjects as an independent dataset. The first ten principal components of population structure, the number of non-missing SNPs used for scoring and inbreeding coefficient for 136 normal subjects were obtained using PLINK. AD PRS based on AD-associated SNPs, including the *APOE* region, was used to predict Ch4 volume in 136 normal subjects. According to the PRS results (Supplementary Figures S5, S6), the best threshold for PRS model is 0.0428, the *p*-value of the PRS model is 0.001. Therefore, AD PRS is related to the Ch4 volume in 136 normal subjects. As for the independent dataset (29 normal subjects), we also obtained the first ten principal components of population structure, the number of non-missing SNPs used for scoring and inbreeding coefficient by PLINK. We used the PRSice to obtain the PRS results (Supplementary Figures S7, S8). The *p*-value of the best PRS model is 0.00011. So AD PRS is also associated with Ch4 volume in an independent dataset. In other word, the association between AD PRS and Ch4 volume can be replicated in an independent dataset.

In order to further validate the reality of this kind of association, we divided the 136 normal subjects into two equal groups. We took one group and another group as training set and test set, respectively. We utilized PLINK to obtain the first ten principal components of population structure, the number of non-missing SNPs used for scoring and inbreeding coefficient for training set and test set, respectively. The PRS results for the training set is showed as (Supplementary Figures S9, S10). The best cutoff for PRS model is 0.05 and the *p*-value of the PRS model is 0.015. According to the PRS results for the test set (Supplementary Figures S11, S12), the *p*-value of the best PRS model is 0.028.Therefore, the AD PRS is related to the Ch4 volume in training set and test set.

All in all, AD PRS is associated with the Ch4 volume in normal subjects. Our study presents several limitations. First of all, the sample size is relatively small. ADNI database provides genetic and images data of more than 800 subjects, including normal control subjects, mild cognitive impairment (MCI) subjects and AD patients. In fact, MCI subjects account for a major portion and AD patients constitute a minor percentage. We selected normal controls and AD patients according to the diagnosis information. Accordingly, we obtained the 106 AD patients and 180 normal control subjects after removing poor-quality subjects in this study. Another limitation is that AD patients were not divided into severe, moderate and mild subgroups according to disease severity. That is mainly because subgroups of AD patients cannot be achieved from the ADNI database. In the future studies, we will collect more sample size as possible as we can and categorize the sample into subgroups to explore the relationship between AD PRS and brain-associated endo-phenotypes. It is not only essential but also meaningful for academic studies.

## Ethics Statement

Data used in preparation of this article were obtained from the ADNI database (adni.loni.usc.edu). Summary results data were obtained from the International Genomics of Alzheimer’s Project (IGAP). Gene expression (median TPM) in multiple human tissues were downloaded from Genotype-Tissue Expression (GTEx) database.

## Author Contributions

QJ and YH designed the experiments. WZ and PR downloaded the MRI data from ADNI database. TW, ZH, and YY performed the experiments. All authors contributed to writing, and approved the final manuscript.

## Conflict of Interest Statement

YY employed by company Jiangsu Singch Pharmaceutical Co., Ltd. The remaining authors declare that the research was conducted in the absence of any commercial or financial relationships that could be construed as a potential conflict of interest.

## Footnotes

1 www.adni-info.org

## Supplementary Material

FIGURE S1 Bar plot showing at broad *P* value thresholds for AD PRS, including the *APOE* region, predicting Ch4 volume in AD patients, including a bar for the best-fit PRS from the high-resolution run.

FIGURE S2 High-resolution plot for AD PRS, including the *APOE* region, predicting Ch4 volume in AD patients. The thick line connects points at the broad *P* value thresholds of Supplementary Figure S1. The best-fit PRS is at *P*_T_ of 0.0068.

FIGURE S3 Bar plot showing at broad *P* value thresholds for AD PRS, including the *APOE* region, predicting Ch4 volume in normal control subjects, including a bar for the best-fit PRS from the high-resolution run.

FIGURE S4 High-resolution plot for AD PRS, including the *APOE* region, predicting Ch4 volume in normal control subjects. The thick line connects points at the broad *P* value thresholds of Supplementary Figure S3. The best-fit PRS is at *P*_T_ of 0.0944.

FIGURE S5 Bar plot showing at broad *P* value thresholds for AD PRS, including the APOE region, predicting Ch4 volume in 136 normal subjects, including a bar for the best-fit PRS from the high-resolution run.

FIGURE S6 High-resolution plot for AD PRS, including the APOE region, predicting Ch4 volume in 136 normal subjects. The thick line connects points at the broad *P* value thresholds of Supplementary Figure S5.

FIGURE S7 Bar plot showing at broad *P* value thresholds for AD PRS, including the APOE region, predicting Ch4 volume in 29 normal subjects, including a bar for the best-fit PRS from the high-resolution run.

FIGURE S8 High-resolution plot for AD PRS, including the APOE region, predicting Ch4 volume in 29 normal subjects. The thick line connects points at the broad *P* value thresholds of Supplementary Figure S7.

FIGURE S9 Bar plot showing at broad *P* value thresholds for AD PRS, including the APOE region, predicting Ch4 volume in training set, including a bar for the best-fit PRS from the high-resolution run.

FIGURE S10 High-resolution plot for AD PRS, including the APOE region, predicting Ch4 volume in training set. The thick line connects points at the broad *P* value thresholds of Supplementary Figure S9.

FIGURE S11 Bar plot showing at broad *P* value thresholds for AD PRS, including the APOE region, predicting Ch4 volume in test set, including a bar for the best-fit PRS from the high-resolution run.

FIGURE S12 High-resolution plot for AD PRS, including the APOE region, predicting Ch4 volume in test set. The thick line connects points at the broad *P* value thresholds of Supplementary Figure S11.

TABLE S1 The neuroimage ID of 106 AD patients.

TABLE S2 The neuroimage ID of 180 NC subjects.

TABLE S3 8070 SNPs with *P* value below 0.0944.
