# Supplementary figures and images for: Polygenic Risk Score for Alzheimer’s Disease Is Associated With Ch4 Volume in Normal Subjects
Source: Front Genet. 2019 Jul 10;10:519. doi: 10.3389/fgene.2019.00519 (PMC6636399; doi:10.3389/fgene.2019.00519)

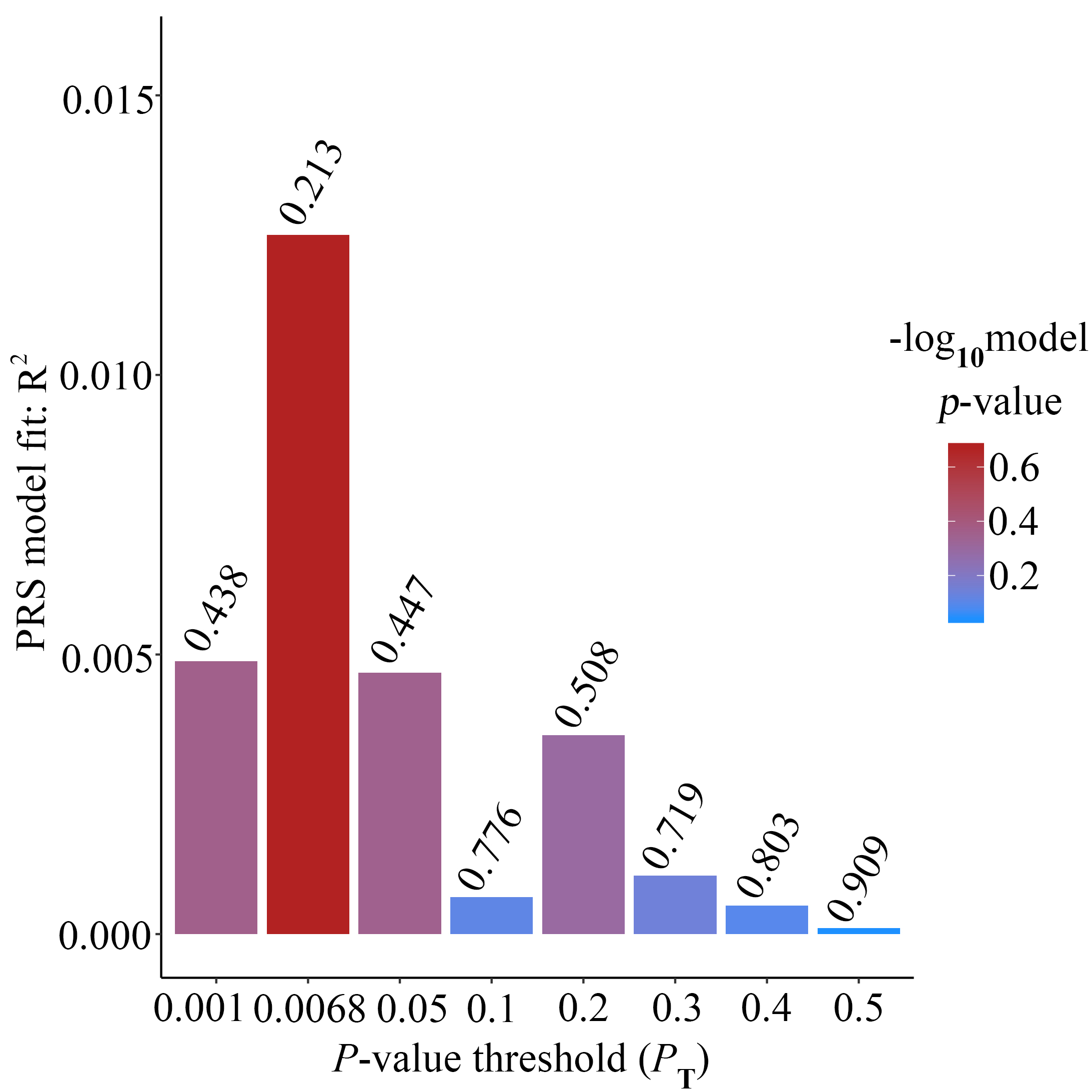

Supplement: FIGURE S1 — Bar plot showing at broad P value thresholds for AD PRS, including the APOE region, predicting Ch4 volume in AD patients, including a bar for the best-fit PRS from the high-resolution run. [file Image_1.JPEG]

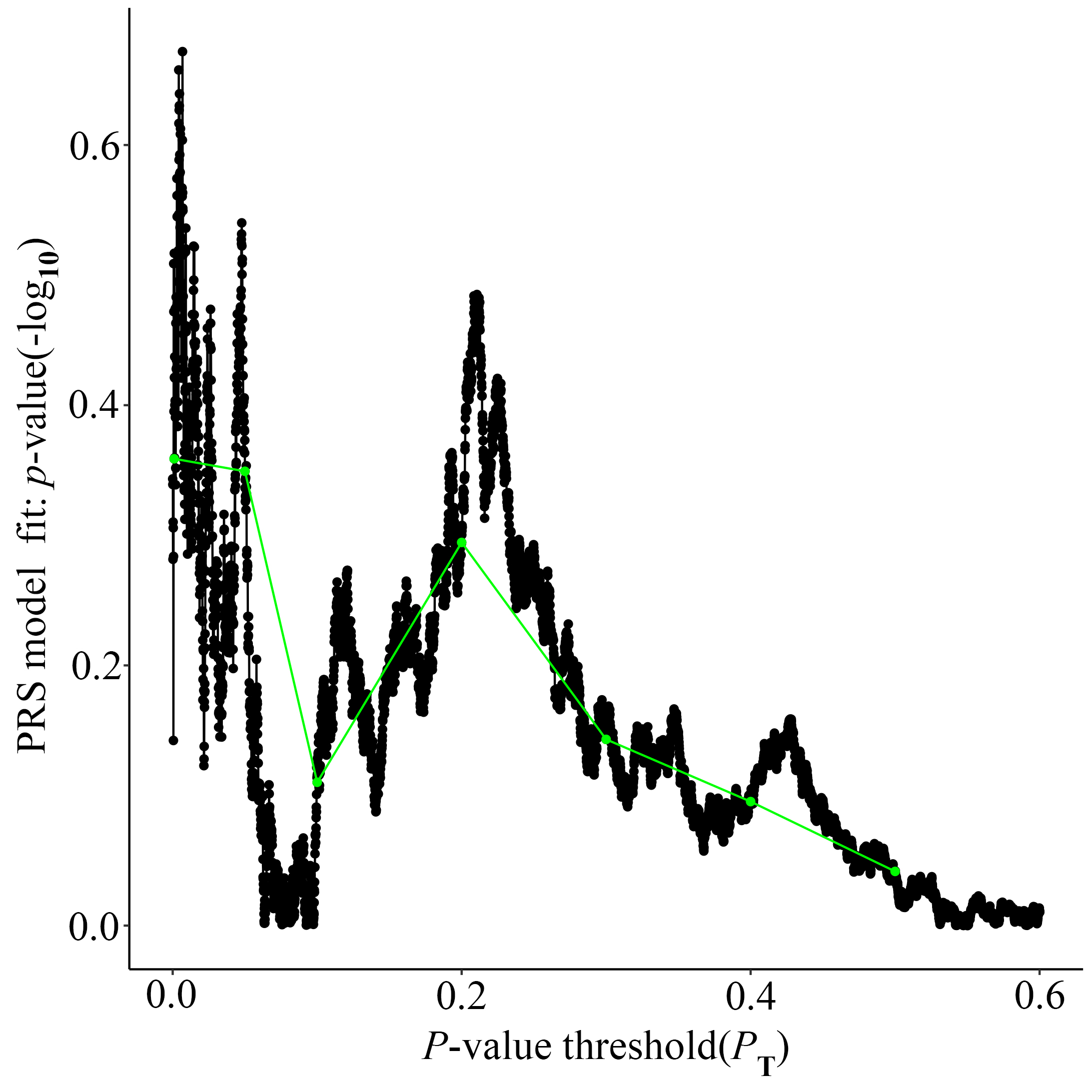

Supplement: FIGURE S2 — High-resolution plot for AD PRS, including the APOE region, predicting Ch4 volume in AD patients. The thick line connects points at the broad P value thresholds of Supplementary Figure S1. The best-fit PRS is at PT of 0.0068. [file Image_2.JPEG]

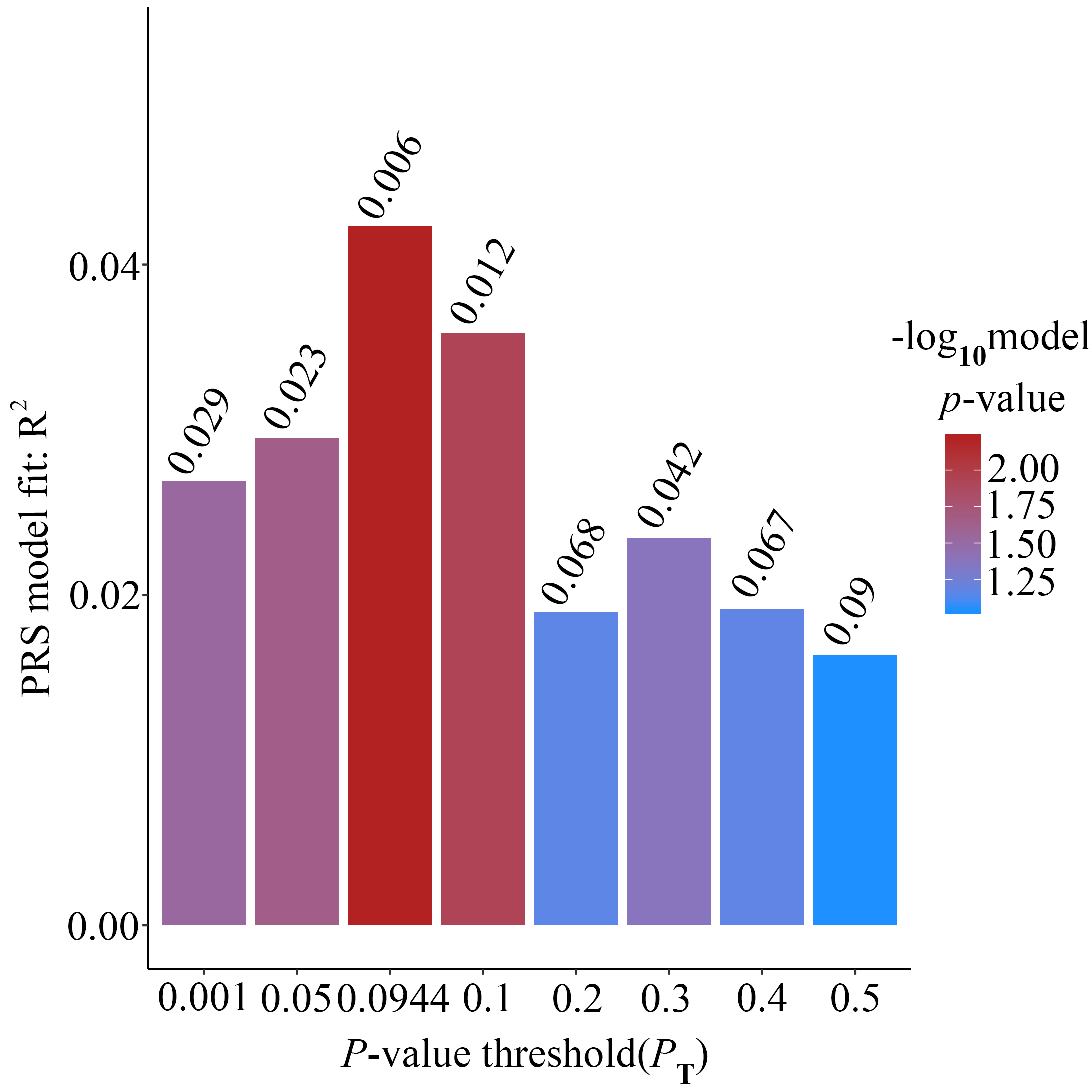

Supplement: FIGURE S3 — Bar plot showing at broad P value thresholds for AD PRS, including the APOE region, predicting Ch4 volume in normal control subjects, including a bar for the best-fit PRS from the high-resolution run. [file Image_3.JPEG]

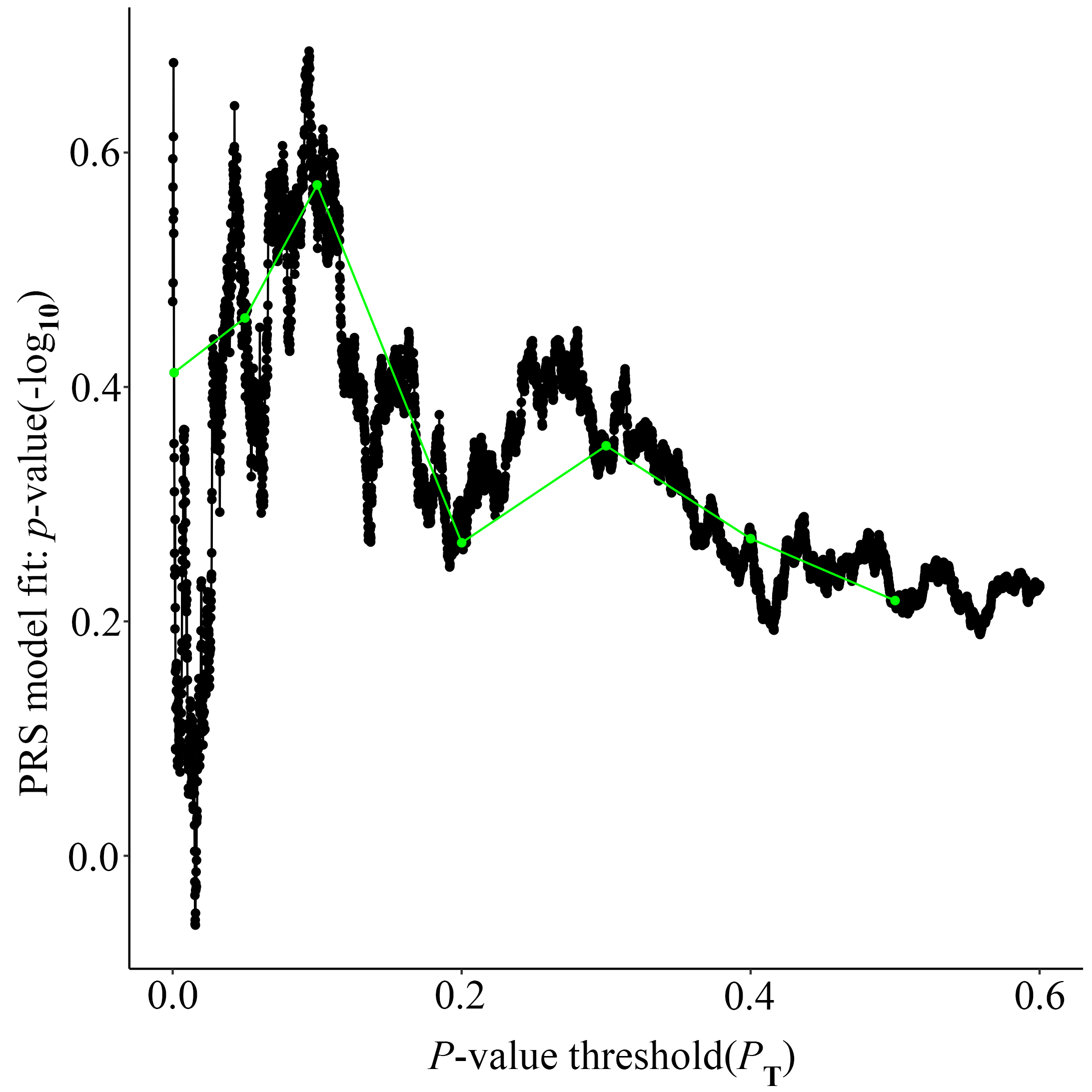

Supplement: FIGURE S4 — High-resolution plot for AD PRS, including the APOE region, predicting Ch4 volume in normal control subjects. The thick line connects points at the broad P value thresholds of Supplementary Figure S3. The best-fit PRS is at PT of 0.0944. [file Image_4.JPEG]

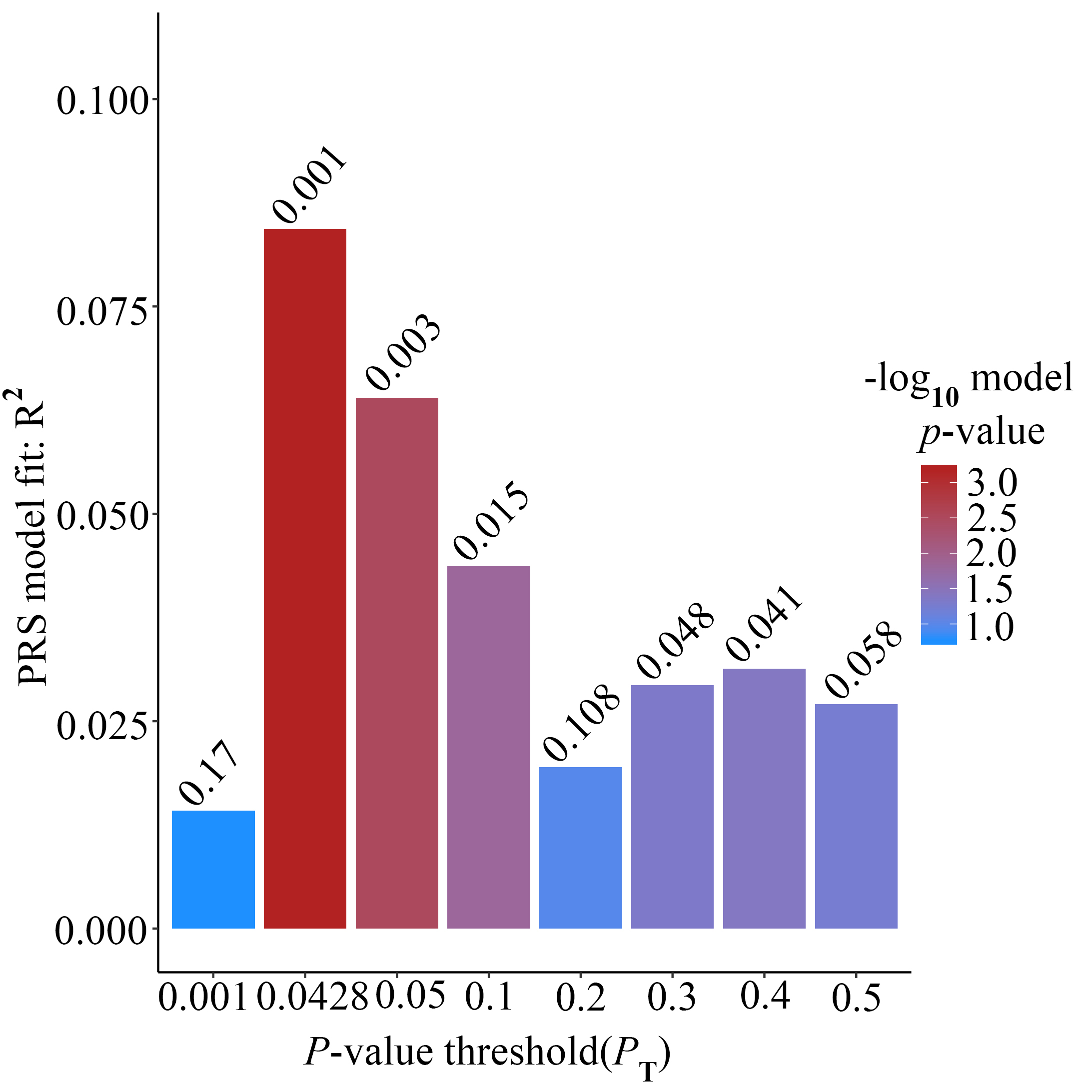

Supplement: FIGURE S5 — Bar plot showing at broad P value thresholds for AD PRS, including the APOE region, predicting Ch4 volume in 136 normal subjects, including a bar for the best-fit PRS from the high-resolution run. [file Image_5.JPEG]

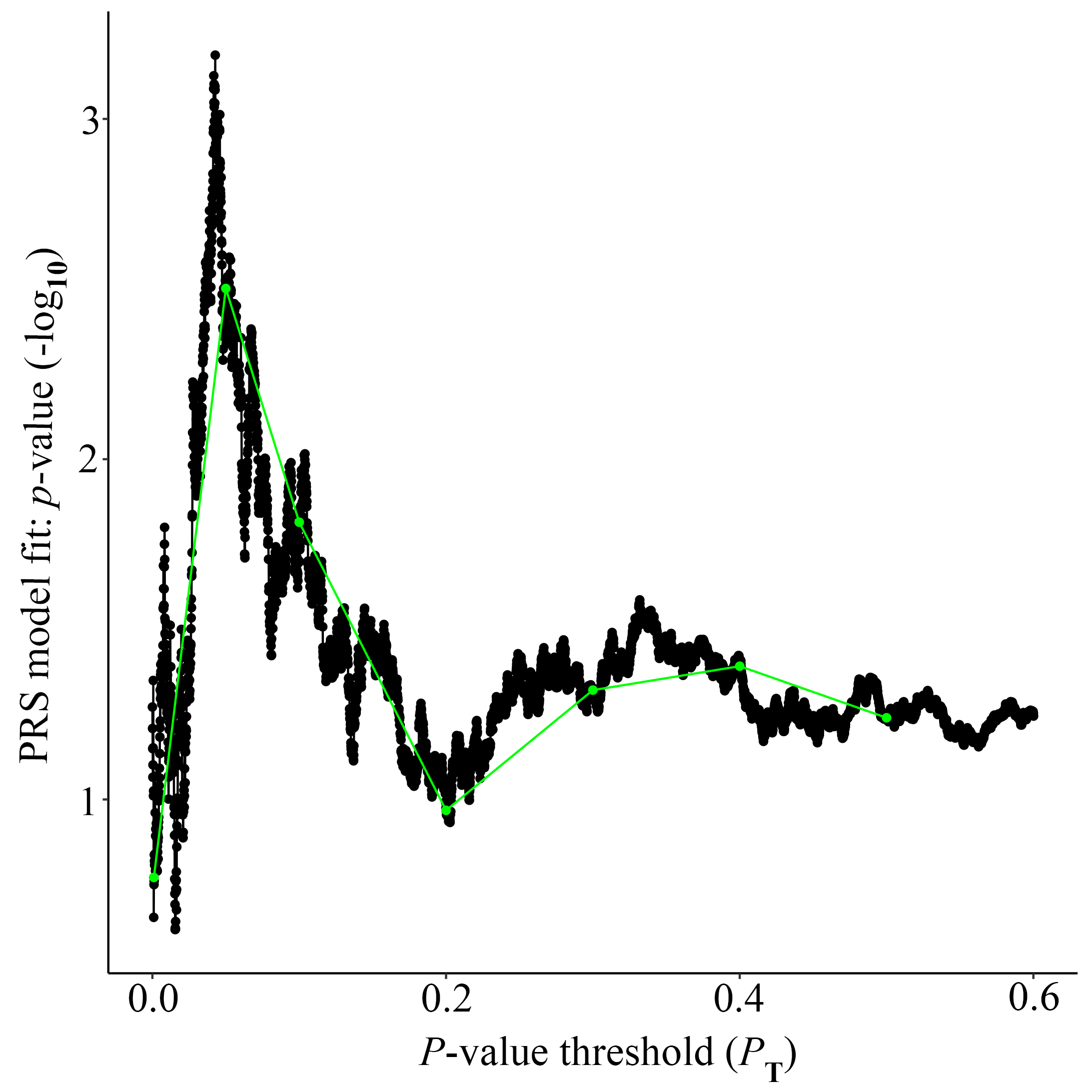

Supplement: FIGURE S6 — High-resolution plot for AD PRS, including the APOE region, predicting Ch4 volume in 136 normal subjects. The thick line connects points at the broad P value thresholds of Supplementary Figure S5. [file Image_6.JPEG]

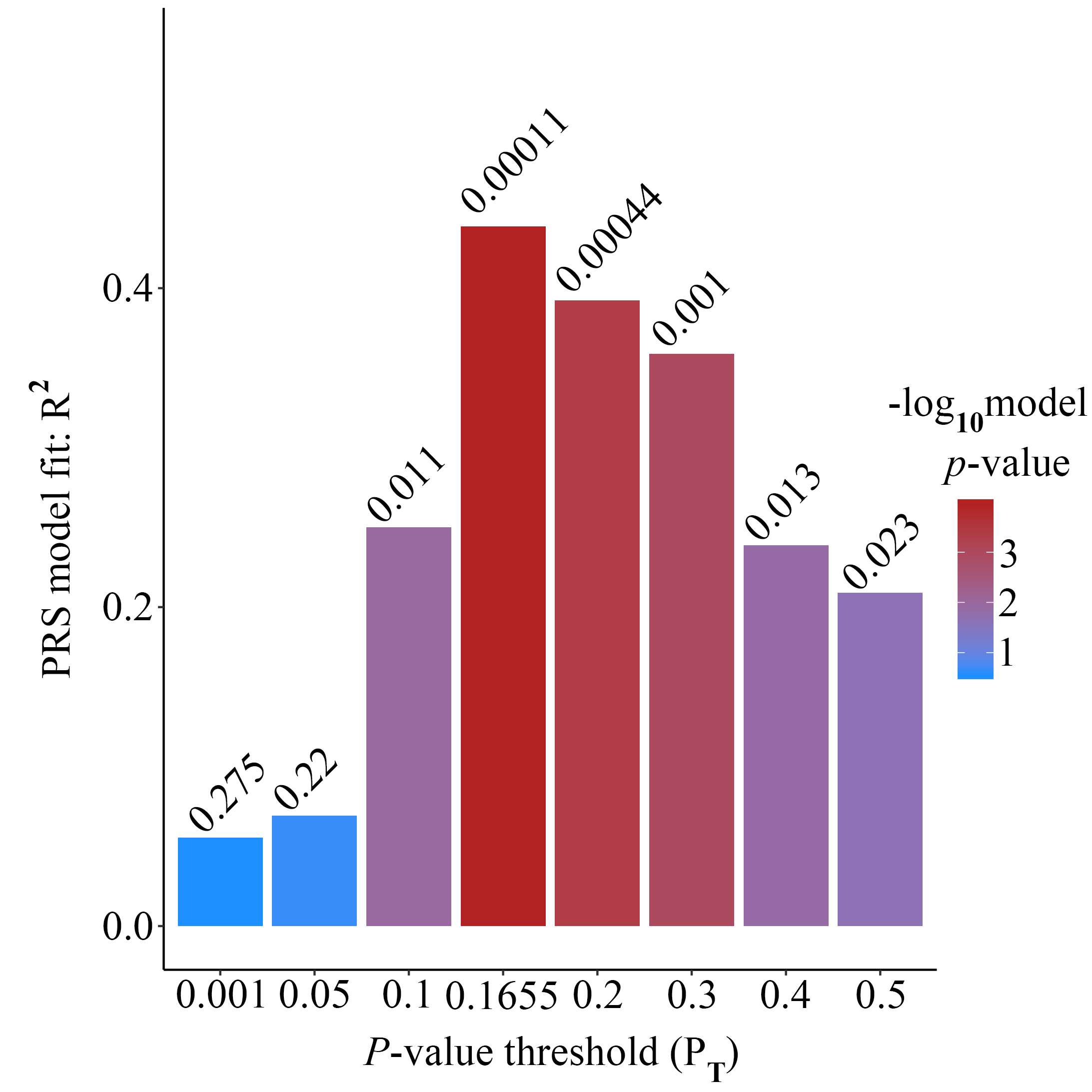

Supplement: FIGURE S7 — Bar plot showing at broad P value thresholds for AD PRS, including the APOE region, predicting Ch4 volume in 29 normal subjects, including a bar for the best-fit PRS from the high-resolution run. [file Image_7.JPEG]

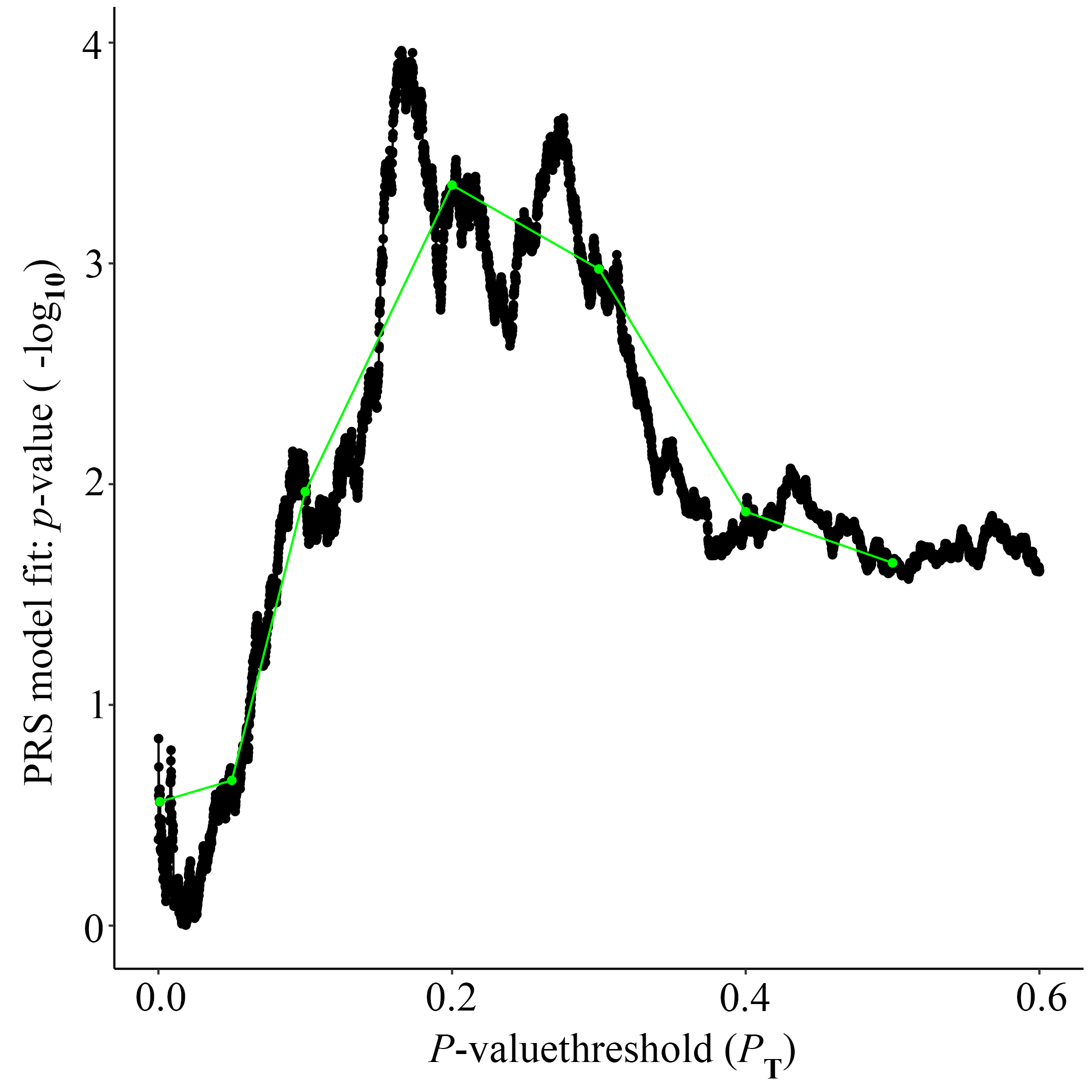

Supplement: FIGURE S8 — High-resolution plot for AD PRS, including the APOE region, predicting Ch4 volume in 29 normal subjects. The thick line connects points at the broad P value thresholds of Supplementary Figure S7. [file Image_8.JPEG]

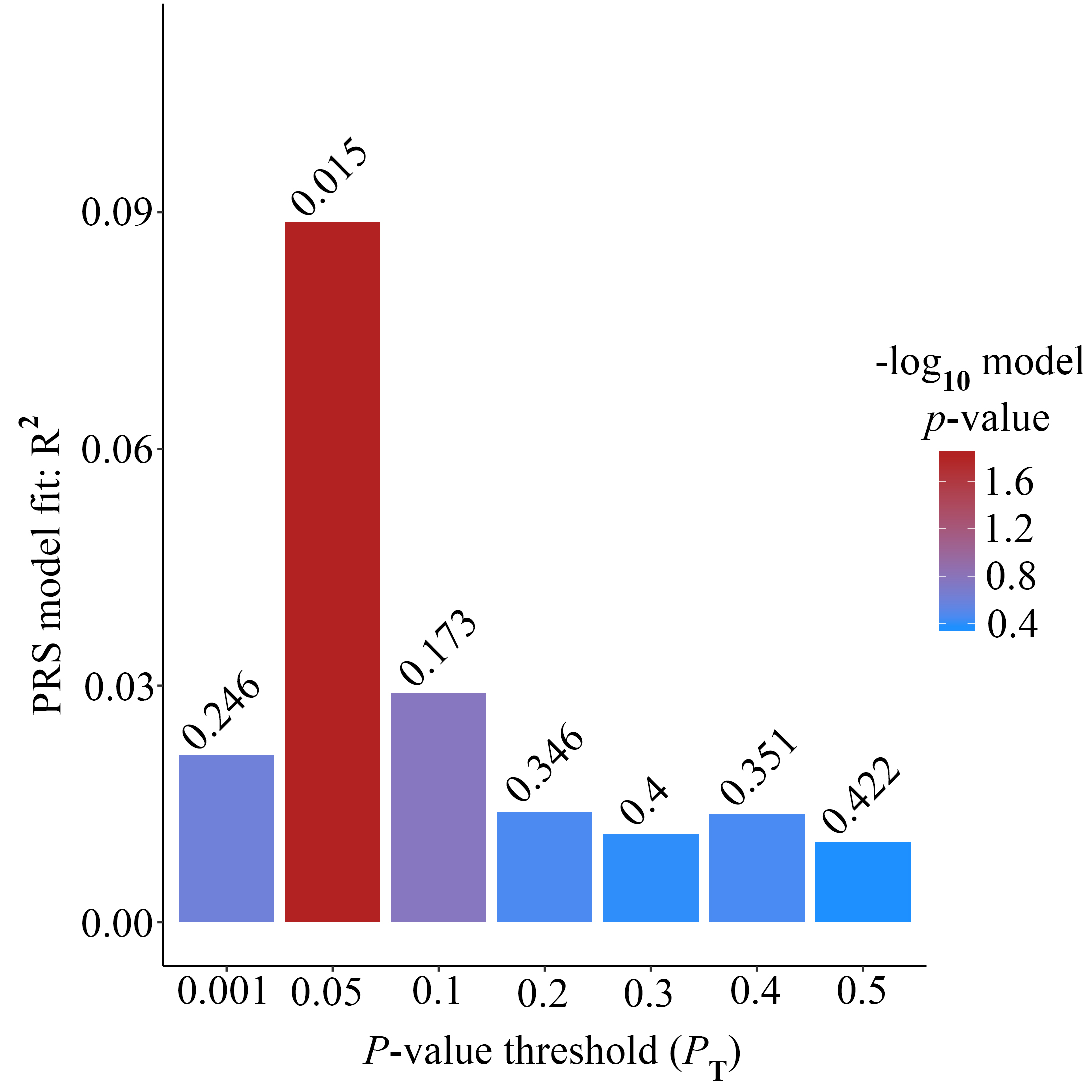

Supplement: FIGURE S9 — Bar plot showing at broad P value thresholds for AD PRS, including the APOE region, predicting Ch4 volume in training set, including a bar for the best-fit PRS from the high-resolution run. [file Image_9.JPEG]

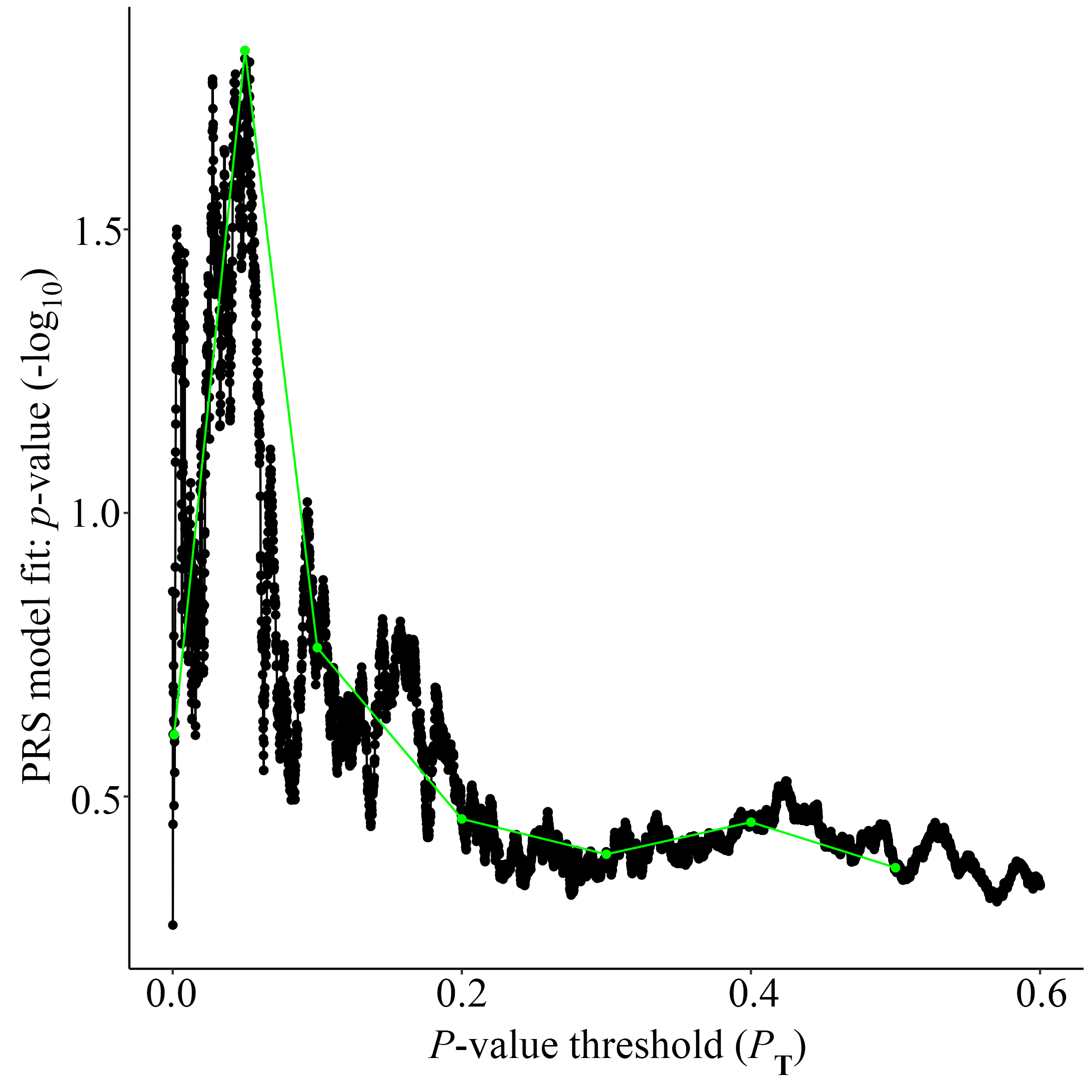

Supplement: FIGURE S10 — High-resolution plot for AD PRS, including the APOE region, predicting Ch4 volume in training set. The thick line connects points at the broad P value thresholds of Supplementary Figure S9. [file Image_10.JPEG]

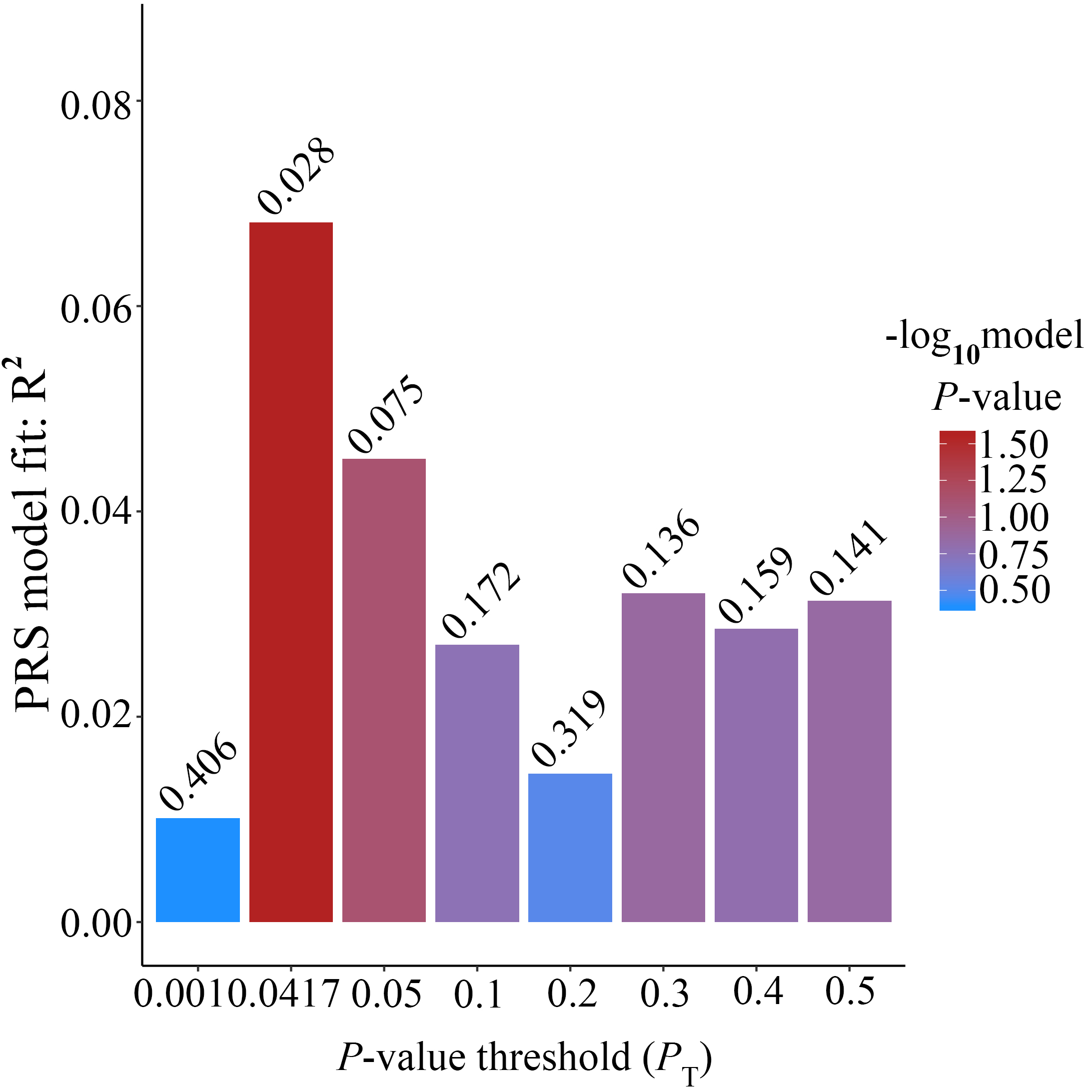

Supplement: FIGURE S11 — Bar plot showing at broad P value thresholds for AD PRS, including the APOE region, predicting Ch4 volume in test set, including a bar for the best-fit PRS from the high-resolution run. [file Image_11.JPEG]

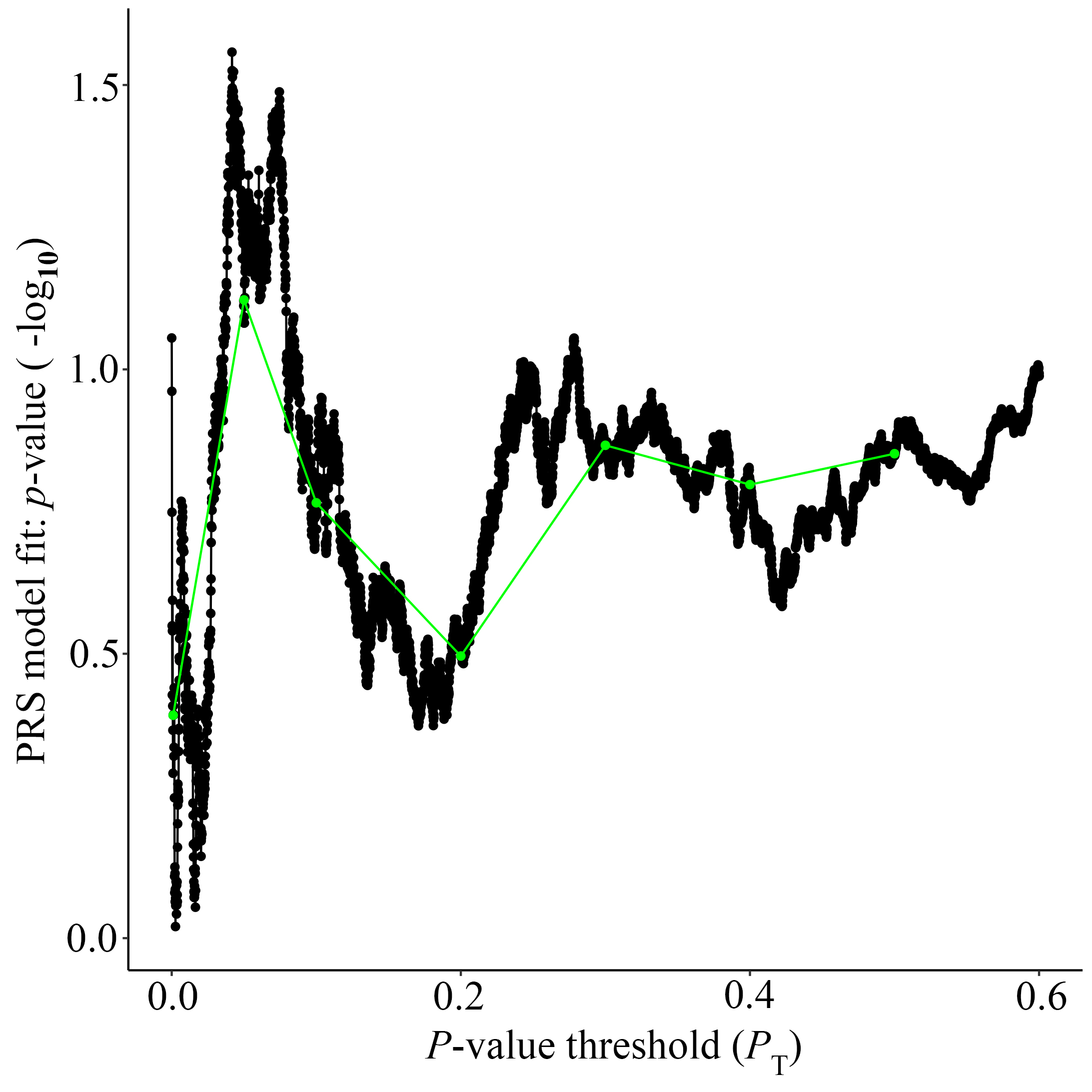

Supplement: FIGURE S12 — High-resolution plot for AD PRS, including the APOE region, predicting Ch4 volume in test set. The thick line connects points at the broad P value thresholds of Supplementary Figure S11. [file Image_12.JPEG]
